# District decision-making for health in low-income settings: a case study of the potential of public and private sector data in India and Ethiopia

**DOI:** 10.1093/heapol/czw017

**Published:** 2016-09-01

**Authors:** Sanghita Bhattacharyya, Della Berhanu, Nolawi Taddesse, Aradhana Srivastava, Deepthi Wickremasinghe, Joanna Schellenberg, Bilal Iqbal Avan

**Affiliations:** ^1^Public Health Foundation of India, Plot No. 47, Sector 44, Gurgaon, 122002, India,; ^2^IDEAS Project, London School of Hygiene and Tropical Medicine, UK and; ^3^JaRco Consulting PLC, Addis Ababa, Ethiopia, PO Box 43107

**Keywords:** Health system blocks, HMIS, private sector, public sector, India, Ethiopia

## Abstract

Many low- and middle-income countries have pluralistic health systems where private for-profit and not-for-profit sectors complement the public sector: data shared across sectors can provide information for local decision-making. The third article in a series of four on district decision-making for health in low-income settings, this study shows the untapped potential of existing data through documenting the nature and type of data collected by the public and private health systems, data flow and sharing, use and inter-sectoral linkages in India and Ethiopia. In two districts in each country, semi-structured interviews were conducted with administrators and data managers to understand the type of data maintained and linkages with other sectors in terms of data sharing, flow and use. We created a database of all data elements maintained at district level, categorized by form and according to the six World Health Organization health system blocks. We used content analysis to capture the type of data available for different health system levels. Data flow in the public health sectors of both counties is sequential, formal and systematic. Although multiple sources of data exist outside the public health system, there is little formal sharing of data between sectors. Though not fully operational, Ethiopia has better developed formal structures for data sharing than India. In the private and public sectors, health data in both countries are collected in all six health system categories, with greatest focus on service delivery data and limited focus on supplies, health workforce, governance and contextual information. In the Indian private sector, there is a better balance than in the public sector of data across the six categories. In both India and Ethiopia the majority of data collected relate to maternal and child health. Both countries have huge potential for increased use of health data to guide district decision-making.

Key messages

- Using a novel application of content analysis, we documented the nature and type of data collected by the public and private health systems, data flow and sharing, and inter-sectoral linkages in India and Ethiopia.
- Ethiopia has better developed formal structures for data sharing than India. In the Indian private sector there is a better balance of data as categorized across the six WHO health system blocks than in the public sector.
- In both India and Ethiopia the majority of data collected by the public and private health systems relate to maternal and child health.

## Introduction

Good quality data from all relevant sources at district level play a major role in health system strengthening. According to the World Health Organization (WHO), attributes of health systems include health services, workforce, supplies, financing, governance and health information (WHO 2007). Out of these six building blocks, data from health information systems can help in planning for health workforce, commodities and infrastructure needed to deliver services that improve health outcomes (WHO 2008).

In many developing countries pluralistic health systems exist where private for-profit and not-for-profit sectors have emerged as important providers of health and key partners in complementing and supplementing the public sector (Haque 2002; Mills *et al.* 2002; WHO 2006; Ejaz *et al.* 2011). Within the public system there are also multiple sectors other than health departments that deliver health services. Coordination between these sectors can save time and improve quality and cost efficiency, leading to improved health outcomes (Gragnolati *et al.* 2005; Nutley 2012; Pelletier *et al.* 2012; Prasad *et al.* 2013).

Strategic and coordinated service delivery within and between the public and private sectors require sharing of information: information shared across sectors can provide comprehensive information for local decision-making, repositioning health service delivery in congruence with the available resources and community health needs (Victora *et al.* 2011).

In low- and middle-income countries, data are not optimally used for routine planning, monitoring and evaluation by the private or public sectors (Ronveaux *et al.* 2005; Gething *et al.* 2006; Lim *et al.* 2008). This is due to: the lack of sharing of complete, accurate and timely data; duplicate and parallel reporting channels; and insufficient capacity to analyse and use data for decision-making (WHO 1997; Garrib *et al.* 2008; Mate *et al.* 2009; Mutale *et al.* 2013). In India (a middle-income country) and Ethiopia (a low-income country), both the public and private sectors play a major role in providing health service delivery, and health data are generated from multiple sources at district level. Though similar in this regard, the district level health systems do vary, most notably in their size, share of public vs private health sector service provision and planning. In India, the private sector accounts for about 80% of outpatient treatment and 60% of hospitalizations (National Sample Survey Organisation, 2004), with 78% of the total health expenditure going towards the private health sector (National Health accounts, 2004–05). Nationally, of women who had a live birth in the 5 years to 2005–06, 21% gave birth in a private health facility and 19% in a public sector one (Pomeroy *et al.* 2010). In contrast, 77% of outpatient care in Ethiopia is provided by the public sector. For-profit and not-for-profit private sector utilization rates are 20 and 1%, respectively (Ethiopia Federal Ministry of Health, 2014). Of the women in Ethiopia who had a live birth in the 5 years to 2005, 5% gave birth in a public sector facility and 0.3% in a private facility (Pomeroy *et al.* 2010). Both countries also have decentralized health systems enabling the assessment of how data are shared for planning at district level. However, their planning structures are different. In India the planning process is bottom-up, whereas in Ethiopia a top-down bottom-up approach is used, allowing for district level planning against a backdrop of national targets. In this context it is important to understand, in both countries, health data flow, sharing and maintenance at different health system levels as well as inter-sectoral^1^ linkages. This process provides insight on how the two countries have adapted their health information systems according to their respective context.

This is the third article in a series of four on the district data for decision-making for health in low-income settings: the first reports the feasibility of establishing a data-informed platform for health to support district data for decision-making in India, Nigeria and Ethiopia; the second reports a systematic literature review of the use of district data for decision-making in low-income settings; and the final article in the series presents prospects for engaging the private sector in health data sharing and collaborative decision-making at district level in India (Avan *et al.* 2016, Wickremasinghe *et al.* 2016, Gautham *et al.* 2016).

Here, we report the nature and type of data collected by public and private health systems, data flow sharing, usage and the inter-sectoral linkages of health data in India and Ethiopia, with a focus on Maternal and Child Health (MCH) data. The unit of analysis is the district, which is the lowest formal unit of administration. In Ethiopia this unit is known as ‘woreda’, but in this article it will be referred to as district.

## Methods

### Study area and health system structure

In India, the central Government is mainly responsible for developing national standards, and sponsoring key programmes while health is a state subject and the state holds primary responsibility for healthcare delivery. The district acts as a link between the state and the local health centres, and is responsible for coordinating with state governments for programme implementation. The service delivery structure in a district comprises primary and community health centres at sub-district level and the sub-centre facility and community level workers at the community level. Through the health sector reform programme the National Rural Health Mission [later renamed the National Health Mission (NHM)] has sought to decentralize planning and increase community involvement, particularly planning and decision-making at district level. Accordingly, a District Programme Management Unit (DPMU) monitors and supports health programmes, collates data and makes plans and budgetary allocation (Ministry of Health and Family Welfare 2006). The NHM further aims to integrate district health plans with those of other sectors such as water, sanitation and nutrition, and to include partnership with non-governmental organizations and coordination with the private health sector (NRHM Division 2007; Ministry of Health and Family Welfare 2012; Prasad *et al.* 2013).

The Ethiopian Government has also taken measures to decentralize the health care system (Earth Institute at Colombia University and Center for National Health Development in Ethiopia). The process of decision-making for health programme development and implementation is shared between the Federal Ministry of Health and the Regional Health Bureaus (RHBs), which also manage policy matters and provide technical support. Zonal Health Departments support the RHBs and District Health Offices in the management of health service delivery, while the District Health Offices are also tasked to manage and coordinate the operation of the primary health care services (Federal Ministry of Health, Ethiopia website). Health services at district level are delivered through Primary Health Care Units (PHCUs). Each PHCU is comprised of one health centre and five satellite health posts. These local health needs are determined through a district-based planning system where the objective is to meet the local health needs within the context of national targets. Health budgets are allocated by the governing body; the District Cabinet, which is responsible for dividing the district budget among different sectors including health, education and agriculture.

### Study area description

The study was undertaken in Sitapur and Unnao districts in Uttar Pradesh, India and in Dendi district in Oromia region and Basso district in Amhara region in Ethiopia (IDEAS 2012a,b). Districts were selected in consultation with NHM representatives in India and Federal Ministry of Health and RHB representatives in Ethiopia, and based on variability in the functioning of health facilities and district health administration, which can have an effect on linkages with different sectors and also the nature and type of health data they maintained.

### Data collection

We sought state (regional in the Ethiopian context) and zonal government support to facilitate visits to health facilities for meetings with key staff. We conducted an initial scoping visit to meet key informants in the public and private sectors in each district, identified on the basis of their role, knowledge and relevance in terms of managing health data. The team visited both strong and weak facilities, determined by the government representatives, at every level of service delivery, to solicit their cooperation. At this stage we outlined the structure of the health system, linkages between central, state (regional) and district levels and the various non-health departments and ministries in operation. After the scoping visit, data collection was conducted between June and September 2012. In India, we visited eight public health facilities at primary and secondary care levels and in Ethiopia we visited eight public health facilities at the primary care level. A complete listing of private sector organizations, both for-profit and not-for-profit, working on MCH in the selected districts was carried out and from that three private sector organizations in Ethiopia and four in India were included as case studies from the two countries. Private sector organizations were selected with the assistance of the district level health offices, using the selection criteria of having a district level office, a registered license to operate and a major presence in the community. At each selected facility we interviewed administrative heads and data managers, in all 35 respondents in Ethiopia and 18 respondents in India. Semi-structured interview guides were used to understand the structure and functions of the organizations, their activities and the type of data collected and maintained, the use of data for preparing district health plans, and linkages with the other sectors in terms of data sharing and flow. The team collected templates of all the data forms that the facility maintained, both article-based and online.

Ethical approval for the study was obtained from the corresponding author’s institute, the Health Ministry Screening Committee in India, and the Science and Technology Ministry in Ethiopia. Verbal consent was obtained for the interviews.

### Data analysis

A Microsoft Access database was created of all the data forms that are maintained at district level by the public and private health sector. Each data form was given a unique number and was categorized based on its source, level of completion (within the health system) and frequency of reporting. The health system categories were adapted from the WHO framework of health system building blocks (WHO 2007). Thematic areas were first identified (e.g. immunization, human resources and expenditure) and sorted into one of the WHO health system categories. Each data element from the collected forms was then categorized according to thematic area (Table 1). Content analysis of the data elements in each form was conducted to capture the type of data available for different health system levels, the level of data sharing and the flow (Weber 1990). An in-depth analysis was done to understand the MCH service delivery data and distal services affecting MCH outcomes such as nutrition, water and sanitation, family planning and abortion care.

**Table 1.** Framework for health system data.

Health system categories	Thematic areas
1. Service delivery	Antenatal care, delivery, postnatal care, newborn care, immunization of infants and children, early childhood development, abortion, family planning, adolescent health, nutrition, water and sanitation, non-communicable disease, communicable disease, TB, malaria, HIV, mortality and morbidity.
2. Contextual factors	Infrastructure of facilities, village and household infrastructure, demography
3. Medical supplies	Resources/supplies
4. Workforce	Human resources, training
5. Governance	Management (supervision), grievance redress, utilization data
6. Finance	Expenditure, financial incentive, insurance scheme

## Results

This section shows the nature and type of data collected by the public and private health systems, data sharing, data flow, use and inter-sectoral linkages of health data at district level in India and Ethiopia.

### Nature and type of data

In both countries’ health systems, data are generated from multiple sources at district level. In the Indian context, multiple departments and ministries outside of the Department of Health also maintain data that are relevant to public health programmes and outcomes. The content analysis of health sector data at district level in India shows that 210 forms are maintained and over 11 810 data elements are collected. In contrast, only 13 forms are maintained by the public and private sectors at district level in Ethiopia, capturing 4287 data elements.

Under the public health system, from community level through to all facility levels, data encompass all attributes of the health system. At district level a wide range of data are collated under each health system category, but the majority pertain to service delivery with limited focus on supplies, health workforce, governance and contextual information (Table 2). In India >50% of the data elements relate to service delivery and under this category data on immunization of infants and children and family planning accounts for the majority. Data on finance and supplies are also adequately maintained at district level in India. Similarly, in Ethiopia three-quarters of the data collected are about service delivery, mainly on immunization of children and family planning.^2^ Eight percent of the data are on resources and supplies, while each of the remaining categories account for <5% of the data elements.

**Table 2.** Nature of data available in district public and private sectors in India and Ethiopia

**Nature of data**	**India**		**Ethiopia**	
	**Total data elements = 11 810**	**Top two categories of data elements**	**Total data elements = 4287**	**Top two categories of data elements**
Service delivery	56%	Child immunization	74%	Child immunization
		Family planning		Family planning
Contextual factors	5%	Demography	5%	Demography
		Health facility infrastructure		Health facility and household infrastructure
Medical supplies	12%	Supplies of medicine and equipment	8%	Supplies of medicine and equipment
Workforce	6%	Availability of staff	5%	Availability of staff
Governance	6%	Management (supervision)	4%	Management (supervision)
		Utilization		Capacity of health facilities
Finance	15%	Expenditure	4%	Expenditure
		Financial incentive		

**Figure 1.**
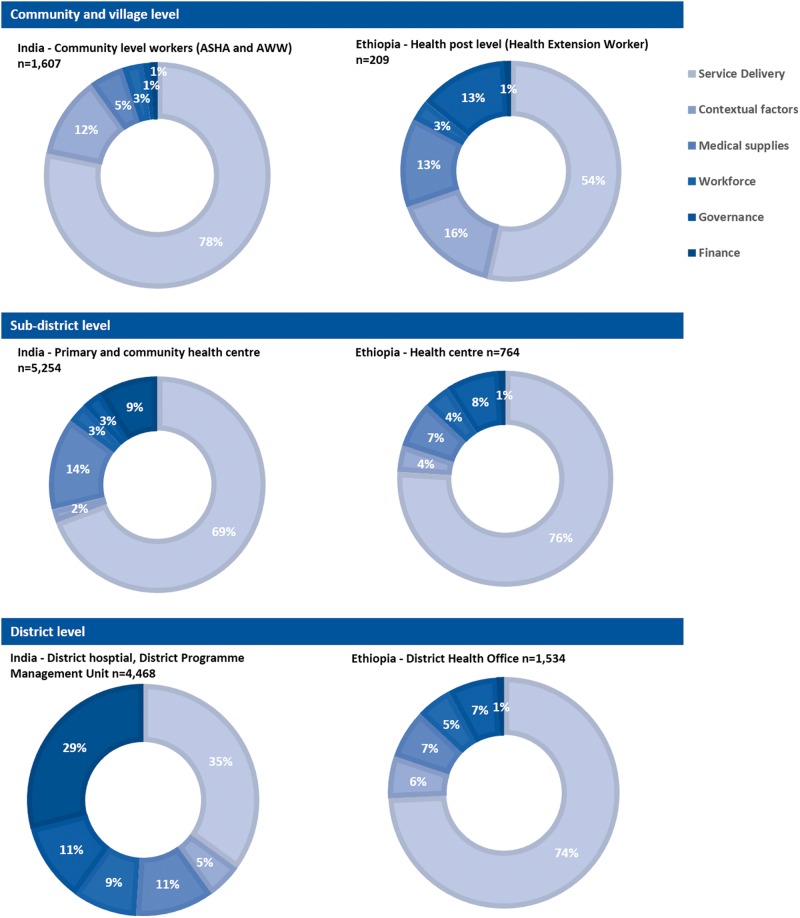
Nature of data available in the district public sector for different levels of the health system in India (*n* = 11 329) and Ethiopia (*n* = 3793). ASHA: Accredited Social Health Activists; AWW: Anganwadi Workers

The distribution of district data maintained at different levels of the public system across the six health categories is shown in Figure 1. In India the greater percentage of the data elements relate to service delivery although, at community level, data pertaining to contextual factors and supplies are also maintained as community health workers conduct an annual survey of the population, infrastructure and supplies of their respective areas. Nevertheless, particularly in terms of contextual information, the information is not collated at higher health system levels. Information about supplies, expenditure and the workforce is primarily maintained at the higher health system level in a district.

In Ethiopia, the majority of data elements are collected at district level (1534), followed by the health centre (764) and then the health post (209). Similar to India, data on service delivery account for the majority of the information collected at all levels of the health system; however, the percentage increases at sub-district and district level. In contrast, at community level, a greater percentage of data are collected on contextual factors, governance and medical supplies, which are key data for assisting the functions of the community health worker. Yet in actual numbers, more data are collected at the sub-district and district levels for each of these categories. At all levels, finance and workforce each make up < 5% of the data elements available.

**Figure 2.**
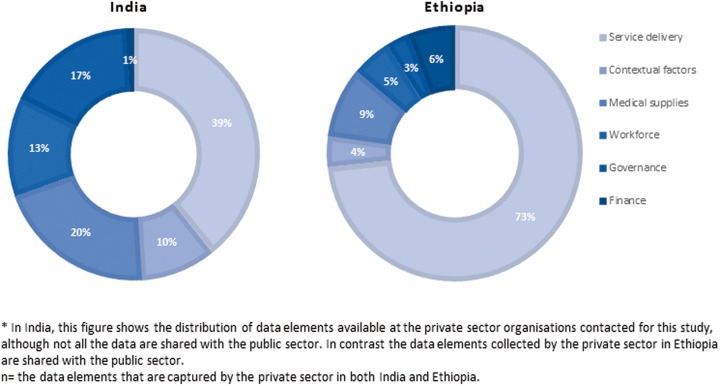
Private sector data elements available at district level in India (*n* = 513) and Ethiopia (*n* = 2732)

The private health sector, including not-for-profit and for-profit organizations, also maintains key health data on service provision (Figure 2). In both countries, the study included only a few for-profit and not-for-profit organizations representing the private health sector. In India, the vast majority of the for-profit private sector is individual service delivery clinics or shops which are not captured in this study.

Based on the few facilities and organizations visited, the private sector in India has fewer data elements than are available in Ethiopia (513 and 2732, respectively). Yet compared with the public sector, with the exception of financial information, there are more data elements available across all the health system categories in the private sector ranging from 10%-20%. Information on finance comprises <1% of the data available. For Ethiopia, the distribution of private-sector data is similar to that of the public system. Seventy-four percent of private sector (for-profit and not-for-profit) data captured are related to service delivery, 9% are about medical supplies and 6% are on finance. Minimal information on governance, finance, workforce and contextual factors is captured.

Among the service delivery data elements, maternal, neonatal and child health programmes account for a major proportion: 46% in India and 27% in Ethiopia. In India, there is a balanced distribution of data across maternal, neonatal and child health programmes (Table 3). In contrast, in Ethiopia over half the data elements are on maternal health with neonatal programmes representing only 3% of available data.

**Table 3.** Distribution of district level MCH service delivery data collected by the public and private sectors

**Categories of MCH service delivery data**	**India**	**Ethiopia**
	**Data elements = 5421**	**Data elements = 1170**
Maternal health	28%	56%
Neonatal health	18%	3%
Child health	34%	27%
Other integrated MCH programme including nutrition, family planning, abortion, sanitation	20%	15%

## Data sharing

The data sharing situation between the public and private sectors is different in the two countries. In India, data from the private health sector (for-profit and not-for-profit) are not practically linked with the public health system, and data sharing is informal and unsystematic. Other than data on institutional deliveries and notifiable diseases such as tuberculosis (TB) and polio, very few data from the private for-profit sector are integrated into the district health management information system (HMIS). As the private for-profit sector needs a once-only registration from the district administration, they are not mandated to share data regularly with the public health system. Similarly the not-for-profit sector shares data only if they are working jointly with public health delivery services. Otherwise, data transfer to the public health system from this sector is also negligible. At the district administrative level (District Magistrate Office—the chief administrator of a district), some convergence can be seen between the public and private sectors but this is neither systematic nor streamlined.

In Ethiopia, a more formal data sharing structure exists between the private and public health system (Figure 4). Private for-profit health facilities are mandated to submit data on HIV, TB and family planning utilization, the number of cases of HIV, TB, malaria and other diseases (communicable and non-communicable) to the District Health Office. The private health facility licenses are renewed on a yearly basis by the District Health Office and as a result submitting reports is essential. Although the HMIS is formatted to collect information from private clinics, the system is not fully operational. The private not-for-profit sector provides quarterly reports to the District Health Officer. Furthermore, as their work is mandated by the government, data on specified HMIS indicators are actively reported to the District Health Office. However, indicators not within HMIS are not reported or utilized for district-level planning.

### Data flow and use

**Figure 3.**
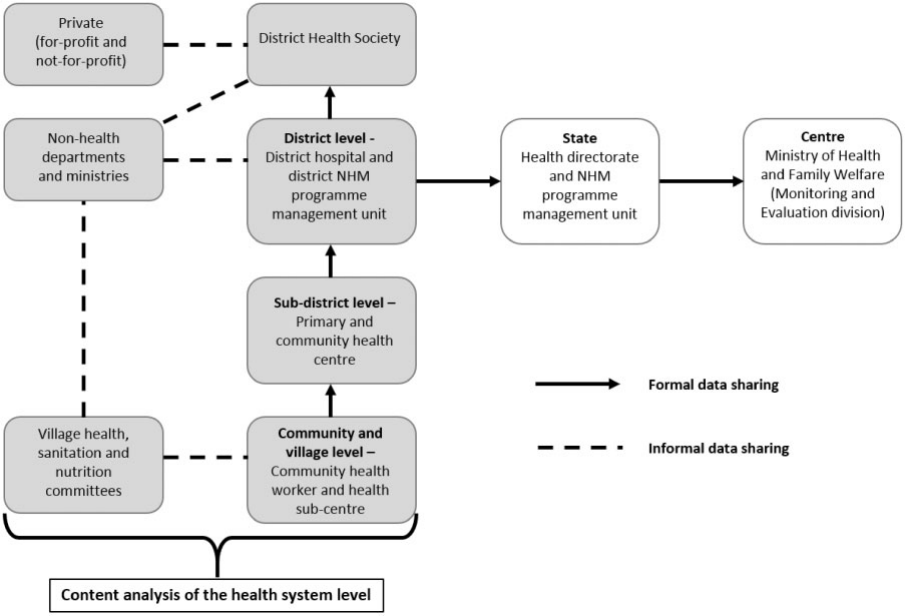
Inter-sectoral linkages in health data flow and sharing in India

In India’s public health system, data flow from the community level upwards (Figure 3). Data from the community and village level, maintained by the community health worker and Auxiliary Nurse Midwife, contain information primarily on service delivery collated on a monthly basis and sent to the sub-district level (primary and community health centres). From here the compiled forms, both article-based and online, are sent to the district level i.e. to the Chief Medical Officer’s office and also to the NHM district programme unit, on a monthly basis. Since the start of the NHM in 2005, there has been a dual health data reporting structure in each district. Facility and community level data are submitted both to the health directorate and the district NHM unit. The district hospital is primarily a service delivery organization and although it is a referral source for primary and community health centres in the district, it does not perform any supervisory functions and does not receive any records from lower levels. Hospitals send data directly to the Chief Medical Officer’s office. The NHM district programme unit further consolidates and verifies the data before sending it to state level. The public health data from the district level are primarily being analysed by the DPMU of the NHM for use when making the annual district programme implementation plan. Data below this level, from facilities and communities, are not being analysed for resource allocation and planning.

**Figure 4.**
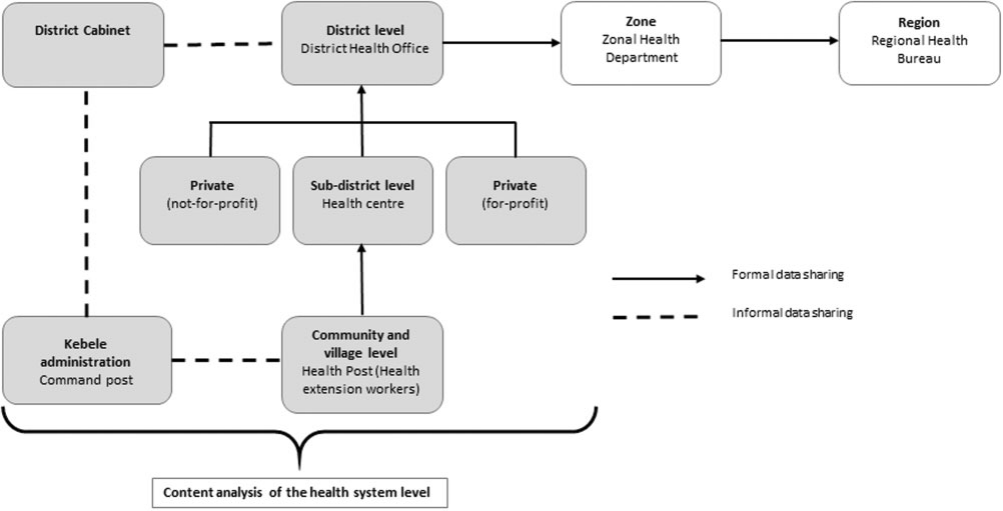
Inter-sectoral linkages in health data flow and sharing in Ethiopia

In terms of the health data flow and use within the public health system of Ethiopia the first level of data collection happens at the health post, which is maintained by the two Health Extension Workers (HEWs). HEWs maintain registers, known as ‘family folders’ on each household and keep records for all preventative packages and some curative services that they provide. Where the family folder is fully operational, it allows HEWs to track and provide follow-up services such as antenatal care, postnatal care and immunization. A summary report is sent as a hard copy to the health centre. At health centre level each department, which includes MCH, HIV, TB, laboratory and pharmacy, maintains a separate register, from which a tally sheet of indicators is submitted on a weekly or monthly basis to the health centre head. Then, the compiled data from the health centre and satellite health posts are sent monthly to the District Health Office. At this level data from all health centres in the district are summed and sent to the Zone Health Department (Figure 4). At zonal level HMIS reports collected from the districts are computerized and the data are sent to the region both as a soft and hard copy.

Information that is gathered by the district public health office is mainly used to assess plans against accomplishments. Information that is collected is also used during the monthly review meetings between the health centre and District Health Office, to give feedback on their performance. Other than this, the data are minimally coordinated, analysed and interpreted at district level for decision-making.

### Inter-sectoral linkages

In India, inter-sectoral coordination has been given particular importance since the launch of the NHM. There are policy guidelines about institutional mechanisms at the central, state and district levels on how better coordination can be fostered with the Departments of Women and Child Development, Rural Development and Panchayati Raj^3^ and Education. The NHM Mission Steering Group, which lays down the policies and programmes for the NHM and includes the Ministers of Health and Family Welfare, Rural Development and Panchayati Raj and Human Resource Development, has been set up at state and district levels in the form of State and District Health Missions. These committees aim to provide a platform for the promotion of policy level convergence, which is yet to be reflected in formal data sharing. Community level coordination with the Department of Women and Child Development can be observed in the joint organization of Village Health and Nutrition Days.^4^ Similarly, the Department of Rural Development and Panchayati Raj is involved in village hygiene and sanitation programmes and the formation of Village Health, Sanitation and Nutrition Committees. However, data sharing across departments is very limited. The Department of Women and Child Development, which is primarily responsible for nutrition programmes, maintains data on child immunization, growth monitoring and child nutrition, but there is no formal data sharing or linkage with the health department. Only at the District Health Society, headed by the District Magistrate, can some level of convergence be seen, where different departments report about their programmes (Figure 3). However, there is no structured format for data reporting and collation and as a result, while annual district programme implementation plans are prepared, data from the non-health sectors are not used to guide resource allocation and planning.

In Ethiopia, aside from the reporting structure that goes from health post to the district health office, inter-sectoral collaboration occurs through forums, which are specific committees at each administrative level that meet to share information and discuss the development sector agenda (Figure 4). The District Cabinet, which is the executive body of the district, makes decisions on the local political administration and basic services delivery. The cabinet meets frequently (weekly) and comprises the heads of each sector (agriculture, education, health, women and child affairs etc.) in the district and is lead by the district administrator. The cabinet discusses progress, particularly in regard to the number of graduated model families, which are families that fulfill all the criteria of development (agriculture, health, education). Cabinet also plans for future activities in each sector. The administrative body for the cabinet is the district council, consisting of elected delegates from each sub-district (*kebele*).

## Discussion

In both India and Ethiopia public health data are collected sequentially, from community to district level, in a formal and systematic manner. District health data are available from multiple sources and have the potential to provide comprehensive information for district level decision-making. Moreover, due to the availability of more data at the district level than at regional, state and national levels in both countries, existing district health data has the potential to shape national policies.

This study was limited to a few districts of India and Ethiopia, and the findings might not be generalizable to other districts in the country. This is particularly so for the private sector, since relatively few private organizations were visited in each district and the selection in India was biased towards larger facilities that regularly maintain records. Other facilities might have more or less data available and may share data with the district public system in a different manner. In contrast, in the public system the data collection forms are uniform across the country. As the main focus of this article was to understand the data content in both the countries, particularly the availability of data elements across the WHO categories, we did not evaluate the adequacy of the data collected, or the reasons behind the non-utilization of data for decision-making. An assessment of data quality in terms of timeliness and accuracy was also not conducted. There are differences in the two countries that it is important to note. The district level population and the volume of private sector service providers in India are much larger than in Ethiopia. However, the study was not designed to compare, but rather to understand how countries adapt the health information system to their specific contexts. Health data available at district level are generic information which is needed for planning and decision-making. Such information is maintained in both countries. The varied contexts in India and Ethiopia helped us to understand the diversity in their practice and also how they have adapted the process. Specifically, the study helped us to understand in two different scenarios how health data are maintained, the linkages and data flow across the sectors.

Comprehensive local health plans depend on adequate local information (WHO 2008; Taghreed and de Savigny 2012). Data from different levels and all the WHO building blocks are key for improving the functioning of the health system and health outcomes (WHO 2007; Nutley 2012). In the study districts, the data collection forms contained information on all six WHO heath system categories and included aspects of governance such as supervisory visits provided and received; targets achieved; number of review meetings organized; timing and quality of data received; problems encountered; and solutions attempted. This reflects the comprehensiveness of the district health data system in both countries. Although the majority of data elements in both countries relate to health service delivery and to MCH, financial, workforce, supply and governance data, which are key to the planning and decision-making process, are regularly collected in the district health system. However, in contrast to Ethiopia, in India data elements pertaining to human resources, finances, governance and supplies are available in the private sector when moving from community up to district level. Further analysis of the data showed that compared with India, minimal information on neonates in Ethiopia was captured.

In low-resource settings, utilization of local health data for planning is often sub-optimal (Nyamtema 2010; Abajebel *et al.* 2011; Akaco *et al.* 2013). The use of data for planning and decision-making is different for the two study countries. In the case of India, the use of data for planning happens at district management unit level, where facility level information is compiled to make the annual district programme implementation plan. Although data are collected at facilities and communities, they are not analysed for resource allocation and planning. Whereas in Ethiopia, information i.e. gathered at the health centres is used to assess plans against accomplishments. The coordination, analysis and interpretation of data at district level for decision-making is limited, and although there are reports of success in data use in the community (see e.g. Azim, 2012; Karim 2015).

In a pluralistic health system, many sectors contribute to achieving a common health outcome. Apart from the health department, other departments and ministries also play a role in providing health services, having a direct or distal effect on health outcomes. Information shared across the sectors can help align the available resources as per a community’s health needs, avoid duplication of efforts and thus help in developing a holistic health plan at district level (Garrib *et al.* 2008; Nyamtema 2010; IDEAS 2012b; Prasad *et al.* 2013).

In both India and Ethiopia, there is little formal data sharing between the private and public health sectors despite multiple sources at district level. This situation is more evident in India, where there is minimal regulation of the private sector by the public sector and it is also not mandatory for the private sector to report to the public health sector. Through the Clinical Establishments Act in 2010, the process of developing legal and regulatory frameworks, which mandate the private sector to share data with public system, has begun. As many states, including the study state, have not adopted the act, private system data do not flow to the district health data system. In Ethiopia, formal reporting structures do exist. Private not-for-profit organizations provide quarterly reports to the district health office and, in theory review meetings should follow the submission to discuss progress, challenges and the way forward. Yet in the districts visited such meetings did not take place due to the small number of not-for-profit organizations. Although the HMIS is designed to capture data from private for-profit organizations, the system is not fully operational. A customized version of the HMIS, that is simplified and less time consuming, might facilitate its use by the private for-profit organizations.

At district level in both countries there is a platform for bringing sectors together: the District Health Society in India and the District Cabinet in Ethiopia. Some information is shared currently, but this lacks coherence and regularity. Although many departments participate in the meeting, there is no structured format through which the different departments share data regularly. The main reason for this is that the departments are from different ministries, where the reporting structure is vertical. The annual district health programme implementation plans are prepared on the basis of data from the health department, not fully incorporating relevant data collected by other departments, which if collated could result in a comprehensive planning and decision-making process.

There are good practices in the two countries which can be adopted for a better health information system. Ethiopia has a structured linkage in place where the private sector and other non-health departments can share information at district level. Comprehensive information is collected in a more concise way, as health data in the public sector are captured using 13 forms. In the Indian public health system, there is a better balance of information at district level across the six WHO building blocks than there is in Ethiopia, particularly better availability of information on financial expenditure. However, more financial information from the private system is needed in both countries. Moreover, to determine the adequacy of data for decision-making at district level, further research is needed to understand the ideal data elements that need to be collected across both public and private health systems.

Health data from multiple sources, if collected in a more structured and regular way, have strong potential for data-based decision-making, which would help to prevent duplication of service delivery. As shown in this study, in a health system where planning takes place at district level, data are available from multiple sources, yet sharing of data is sub-optimal. A mechanism is needed to bring governmental and non-governmental service providers to a common forum on a regular basis and to further strengthen the District Health Society and District Cabinet platforms. The forum can be used to share data in a systematic manner, use information as a tool in priority setting for resource allocation, and needs assessment for further allocation of funds. A well-functioning and coordinated health information system can further strengthen the five building blocks of a health system in order to achieve desired health goals (Evans and Stansfield 2003; WHO 2009; Chan *et al.* 2010; Nutley and Reynolds 2013).

## Conclusion

This study helps to understand how, in two countries with varying contexts, health data are maintained, linked, used and flow within the health system and across different sectors. Health data for all six WHO categories are collected in both India and Ethiopia, indicating that there is a huge potential in both countries for increased use of data to guide district decision-making. When compared with Ethiopia, India’s health information system has a better balance of information across the six building blocks, particularly information generated in the private health sector. In both countries, formal data sharing between the private and public health sectors is minimal, but Ethiopia has a framework in place whereby the private health sector can share information at district level. There is a district level platform in both countries that brings the public and private sectors together. However, for district-level plans to be more holistic and data driven, there is a need to make the platform more structured to create a space for formal data sharing across the two health sectors and with non-health-related sectors.

## Ethical approval

This research was conducted with a letter of support from the Ethiopian Federal Ministry of Health, national approval from the Ministry of Science and Technology in Ethiopia and regional approval from the Independent Ethics Review Board of Ethiopia; approval from the Health Systems Research Division of the Indian Council of Medical Research and the Society for the Promotion of Ethical Clinical Trials, India; and approval from the Research Ethics Committee at the London School of Hygiene and Tropical Medicine. The free and informed consent of all interviewees was obtained.

## Notes

1 The use of the word ‘sector’ in the context of this article relates to the key service providers both public departments or ministries, which have proximal and distal roles in the delivery of health services in a district, and private providers.

2 Ethiopia data has information regarding morbidity and mortality at service provision level, but this mostly pertains to the number of cases rather than the service provided. This has been excluded from the results shown here.

3 Panchayat Raj is a system of governance in which panchayats are the basic units of administration. It has three levels: Gram (village, though it can comprise more than one village), Janpad (taluka or block) and Zilla (district).

4 Village Health and Nutrition Days are organized once a month in a village by the three community health workers, so the villagers can obtain basic services and information. They can also learn about the preventive and promotive aspects of health care, which will encourage them to seek health care at proper facilities.
